# Supplementary material for: Impact of rice GENERAL REGULATORY FACTOR14h (GF14h) on low-temperature seed germination and its application to breeding
Source: PLoS Genet. 2024 Aug 7;20(8):e1011369. doi: 10.1371/journal.pgen.1011369 (PMC11343456; doi:10.1371/journal.pgen.1011369)
Supplement: S1 Fig — (A) Frequency distribution of germination rates at 13°C after eight days from seed imbibition in 200 F7 RILs derived from a cross between Iwatekko and Arroz da Terra [31]. (B) Selection of RILs with low cold germination rates. The 62 RILs with the lower germination rates from the first test (A) were tested for the second, and then the 20 RILs with the lowest germination rates were selected as a bulk sample for QTL-seq analysis. The bar graph shows the mean values of the two tests. (C) Selection of RILs with high cold germination rates. The 37 RILs with the higher germination rates from the first test (A) were tested for the second, and then the 20 RILs with the highest germination rates were selected for a bulk sample for QTL-seq analysis. The bar graph shows the average values of the two tests. (PDF) [file pgen.1011369.s001.pdf]

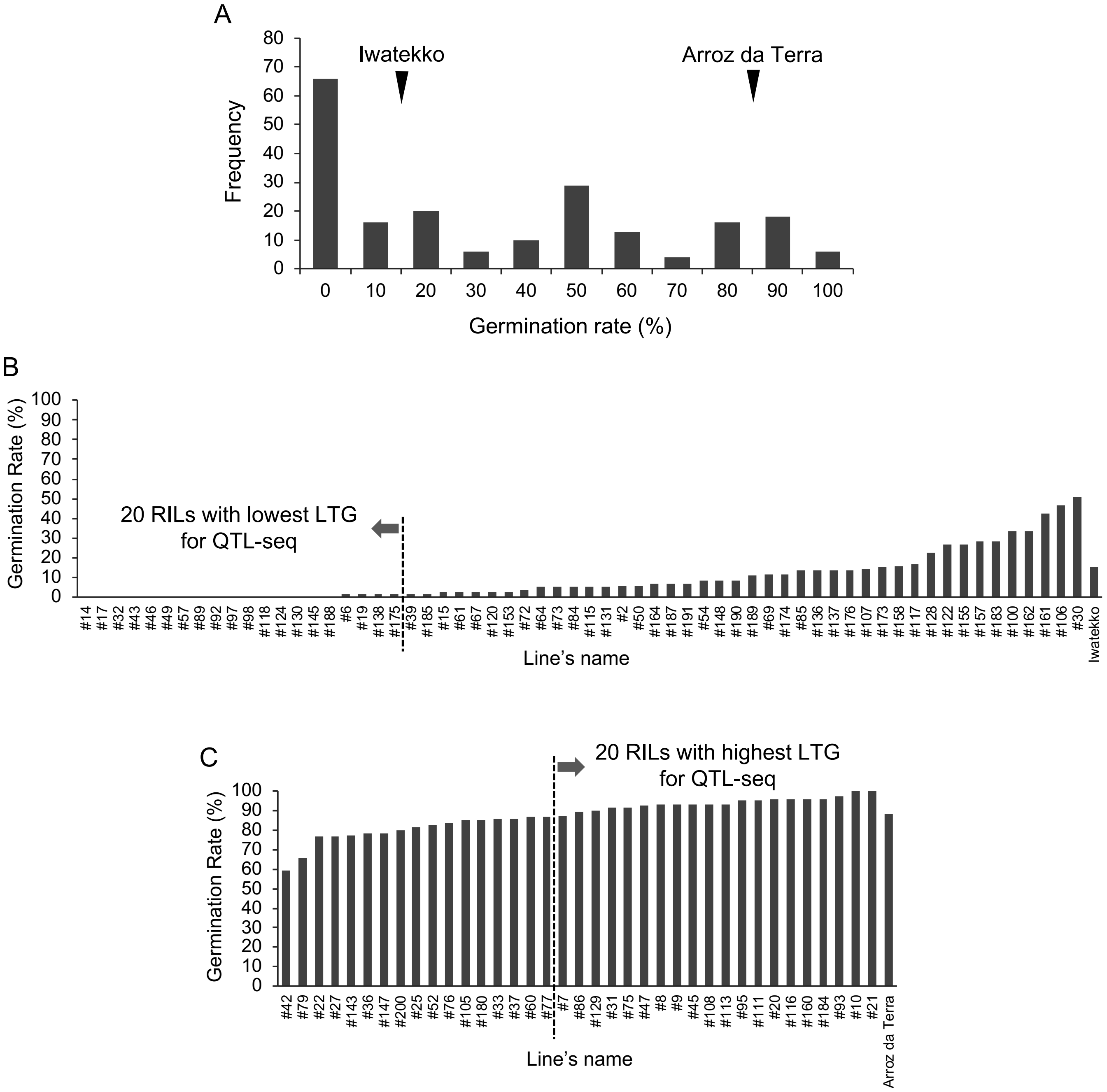

S1 Fig. Frequency distribution of germination rates in the RIL population and germination rates of selected RILs for QTL-seq analysis.

(A) Frequency distribution of germination rates at 13° C after eight days from seed imbibition in 200 F_7 RILs derived from a cross between Iwatekko and Arroz da Terra [31]. **(B)** Selection of RILs with low cold germination rates. The 62 RILs with the lower germination rates from the first test (A) were tested for the second, and then the 20 RILs with the lowest germination rates were selected as a bulk sample for QTL-seq analysis. The bar graph shows the mean values of the two tests. **(C)** Selection of RILs with high cold germination rates. The 37 RILs with the higher germination rates from the first test (A) were tested for the second, and then the 20 RILs with the highest germination rates were selected for a bulk sample for QTL-seq analysis. The bar graph shows the average values of the two tests.
