# Supplementary material for: Impact of rice GENERAL REGULATORY FACTOR14h (GF14h) on low-temperature seed germination and its application to breeding
Source: PLoS Genet. 2024 Aug 7;20(8):e1011369. doi: 10.1371/journal.pgen.1011369 (PMC11343456; doi:10.1371/journal.pgen.1011369)
Supplement: S2 Fig — Arroz da Terra and Italica Livorno harbor a functional qLTG3-1 variant. Nipponbare carries another functional qLTG3-1 variant due to the nonsynonymous substitution (*). Iwatekko, Hitomebore, and Hayamasari contain a loss-of-function variant for qLTG3-1 due to a 71-bp deletion. (PDF) [file pgen.1011369.s002.pdf]

```
                        10        20        30        40        50        60
                                                                *
Arroz da Terra  ATGGCGACGAAAGCTGGGGTGATCGCCACGCTCCTGGCCCTGAACCTCCTCTTCTTCACC
Italica Livorno ATGGCGACGAAAGCTGGGGTGATCGCCACGCTCCTGGCCCTGAACCTCCTCTTCTTCACC
Iwatekko        ATGGCGACGAAAGCTGGGGTGATCGCCACGCTCCTGGCCCTGAACC~~~~~~~~~~~~~~
Hitomebore      ATGGCGACGAAAGCTGGGGTGATCGCCACGCTCCTGGCCCTGAACC~~~~~~~~~~~~~~
Hayamasari      ATGGCGACGAAAGCTGGGGTGATCGCCACGCTCCTGGCCCTGAACC~~~~~~~~~~~~~~
Nipponbare      ATGGCGACGAAAGCTGGGGTGATCGCCACGCTCCTGGCCCTGAACCTCCACTTCTTCACC

                        70        80        90       100       110       120
Arroz da Terra  TTCTCCGACGCGTGCGGCTGCCAGTGCGGCTCATGCCCTAGTCCCGGCGGAGGAGGCGGT
Italica Livorno TTCTCCGACGCGTGCGGCTGCCAGTGCGGCTCATGCCCTAGTCCCGGCGGAGGAGGCGGT
Iwatekko        ~~~~~~~~~~~~~~~~~~~~~~~~~~~~~~~~~~~~~~~~~~~~~~~~~~~~~~~~~GGT
Hitomebore      ~~~~~~~~~~~~~~~~~~~~~~~~~~~~~~~~~~~~~~~~~~~~~~~~~~~~~~~~~GGT
Hayamasari      ~~~~~~~~~~~~~~~~~~~~~~~~~~~~~~~~~~~~~~~~~~~~~~~~~~~~~~~~~GGT
Nipponbare      TTCTCCGACGCGTGCGGCTGCCAGTGCGGCTCATGCCCTAGTCCCGGCGGAGGAGGCGGT

                       130       140       150       160       170       180
Arroz da Terra  GGCGGTGGCGGTGGCGGTGGCGGTGGTGGAGGGCGTGGAGGTGGTGGCGGGAGCGGCGGA
Italica Livorno GGCGGTGGCGGTGGCGGTGGCGGTGGTGGAGGGCGTGGAGGTGGTGGCGGGAGCGGCGGA
Iwatekko        GGCGGTGGCGGTGGCGGTGGCGGTGGTGGAGGGCGTGGAGGTGGTGGCGGGAGCGGCGGA
Hitomebore      GGCGGTGGCGGTGGCGGTGGCGGTGGTGGAGGGCGTGGAGGTGGTGGCGGGAGCGGCGGA
Hayamasari      GGCGGTGGCGGTGGCGGTGGCGGTGGTGGAGGGCGTGGAGGTGGTGGCGGGAGCGGCGGA
Nipponbare      GGCGGTGGCGGTGGCGGTGGCGGTGGTGGAGGGCGTGGAGGTGGTGGCGGGAGCGGCGGA

                       190       200       210       220       230       240
Arroz da Terra  GGTTCAGGTGGAGGCGGCGGTTCAGGTGGAGGAGGAAGCGGCGGCGGAGGTTCAGGCGGT
Italica Livorno GGTTCAGGTGGAGGCGGCGGTTCAGGTGGAGGAGGAAGCGGCGGCGGAGGTTCAGGCGGT
Iwatekko        GGTTCAGGTGGAGGCGGCGGTTCAGGTGGAGGAGGAAGCGGCGGCGGAGGTTCAGGCGGT
Hitomebore      GGTTCAGGTGGAGGCGGCGGTTCAGGTGGAGGAGGAAGCGGCGGCGGAGGTTCAGGCGGT
Hayamasari      GGTTCAGGTGGAGGCGGCGGTTCAGGTGGAGGAGGAAGCGGCGGCGGAGGTTCAGGCGGT
Nipponbare      GGTTCAGGTGGAGGCGGCGGTTCAGGTGGAGGAGGAAGCGGCGGCGGAGGTTCAGGCGGT

                       250       260       270       280       290       300
Arroz da Terra  GGAGGAAGCGGAGGCGGCGGAGGAGGAGGAAGCGGCGGCGGCGGGGGAGGAGGAAGATGC
Italica Livorno GGAGGAAGCGGAGGCGGCGGAGGAGGAGGAAGCGGCGGCGGCGGGGGAGGAGGAAGATGC
Iwatekko        GGAGGAAGCGGAGGCGGCGGAGGAGGAGGAAGCGGCGGCGGCGGGGGAGGAGGAAGATGC
Hitomebore      GGAGGAAGCGGAGGCGGCGGAGGAGGAGGAAGCGGCGGCGGCGGGGGAGGAGGAAGATGC
Hayamasari      GGAGGAAGCGGAGGCGGCGGAGGAGGAGGAAGCGGCGGCGGCGGGGGAGGAGGAAGATGC
Nipponbare      GGAGGAAGCGGAGGCGGCGGAGGAGGAGGAAGCGGCGGCGGCGGGGGAGGAGGAAGATGC

                       310       320       330       340       350       360
Arroz da Terra  CCGATCGACACGCTGAAGCTGGGGGTGTGCGCGAACGTGCTGAATGGGCTGATAAACGTG
Italica Livorno CCGATCGACACGCTGAAGCTGGGGGTGTGCGCGAACGTGCTGAATGGGCTGATAAACGTG
Iwatekko        CCGATCGACACGCTGAAGCTGGGGGTGTGCGCGAACGTGCTGAATGGGCTGATAAACGTG
Hitomebore      CCGATCGACACGCTGAAGCTGGGGGTGTGCGCGAACGTGCTGAATGGGCTGATAAACGTG
Hayamasari      CCGATCGACACGCTGAAGCTGGGGGTGTGCGCGAACGTGCTGAATGGGCTGATAAACGTG
Nipponbare      CCGATCGACACGCTGAAGCTGGGGGTGTGCGCGAACGTGCTGAATGGGCTGATAAACGTG

                       370       380       390       400       410       420
Arroz da Terra  CAGCTGGGGACGCCGCCGCGGCAGCCGTGTTGCAGCCTCATCCAGGGCCTCGCCGACCTT
Italica Livorno CAGCTGGGGACGCCGCCGCGGCAGCCGTGTTGCAGCCTCATCCAGGGCCTCGCCGACCTT
Iwatekko        CAGCTGGGGACGCCGCCGCGGCAGCCGTGTTGCAGCCTCATCCAGGGCCTCGCCGACCTT
Hitomebore      CAGCTGGGGACGCCGCCGCGGCAGCCGTGTTGCAGCCTCATCCAGGGCCTCGCCGACCTT
Hayamasari      CAGCTGGGGACGCCGCCGCGGCAGCCGTGTTGCAGCCTCATCCAGGGCCTCGCCGACCTT
Nipponbare      CAGCTGGGGACGCCGCCGCGGCAGCCGTGTTGCAGCCTCATCCAGGGCCTCGCCGACCTT

                       430       440       450       460       470       480
Arroz da Terra  GAGGCCGCCGTGTGCCTCTGCACCGCCCTCCGCGCCAACATCCTTGGCATCAACCTCAAC
Italica Livorno GAGGCCGCCGTGTGCCTCTGCACCGCCCTCCGCGCCAACATCCTTGGCATCAACCTCAAC
Iwatekko        GAGGCCGCCGTGTGCCTCTGCACCGCCCTCCGCGCCAACATCCTTGGCATCAACCTCAAC
Hitomebore      GAGGCCGCCGTGTGCCTCTGCACCGCCCTCCGCGCCAACATCCTTGGCATCAACCTCAAC
Hayamasari      GAGGCCGCCGTGTGCCTCTGCACCGCCCTCCGCGCCAACATCCTTGGCATCAACCTCAAC
Nipponbare      GAGGCCGCCGTGTGCCTCTGCACCGCCCTCCGCGCCAACATCCTTGGCATCAACCTCAAC

                       490       500       510       520       530       540
Arroz da Terra  CTCCCCATCAACCTCAGCCTCCTCGTCAACTACTGCGGCCGCTCCGTCCCCTCCGGCTTC
Italica Livorno CTCCCCATCAACCTCAGCCTCCTCGTCAACTACTGCGGCCGCTCCGTCCCCTCCGGCTTC
Iwatekko        CTCCCCATCAACCTCAGCCTCCTCGTCAACTACTGCGGCCGCTCCGTCCCCTCCGGCTTC
Hitomebore      CTCCCCATCAACCTCAGCCTCCTCGTCAACTACTGCGGCCGCTCCGTCCCCTCCGGCTTC
Hayamasari      CTCCCCATCAACCTCAGCCTCCTCGTCAACTACTGCGGCCGCTCCGTCCCCTCCGGCTTC
Nipponbare      CTCCCCATCAACCTCAGCCTCCTCGTCAACTACTGCGGCCGCTCCGTCCCCTCCGGCTTC

                       550
Arroz da Terra  CAGTGCAGCAACTAA
Italica Livorno CAGTGCAGCAACTAA
Iwatekko        CAGTGCAGCAACTAA
Hitomebore      CAGTGCAGCAACTAA
Hayamasari      CAGTGCAGCAACTAA
Nipponbare      CAGTGCAGCAACTAA
```

S2 Fig. Multiple DNA sequence alignment of *qLTG3-1* variants.
Arroz da Terra and Italica Livorno harbor a functional *qLTG3-1* variant. Nipponbare carries another functional *qLTG3-1* variant due to the nonsynonymous substitution (*). Iwatekko, Hitomebore, and Hayamasari contain a loss-of-function variant for *qLTG3-1* due to a 71-bp deletion.
