# Supplementary material for: Impact of rice GENERAL REGULATORY FACTOR14h (GF14h) on low-temperature seed germination and its application to breeding
Source: PLoS Genet. 2024 Aug 7;20(8):e1011369. doi: 10.1371/journal.pgen.1011369 (PMC11343456; doi:10.1371/journal.pgen.1011369)
Supplement: S3 Fig — (A) Strategy for the development of qLTG3-2-NIL, qLTG11-NIL, and NIL-GF14hArroz. Molecular markers were used for foreground and background selection. (B) Diagram showing the genotype of NIL-GF14hArroz. NIL-GF14hArroz contains a 172-kb region on chromosome 11 harboring the Arroz da Terra allele of GF14h. Light blue bars indicate genomic fragments from Hitomebore; red bars indicate genomic fragments from Arroz da Terra. (PDF) [file pgen.1011369.s003.pdf]

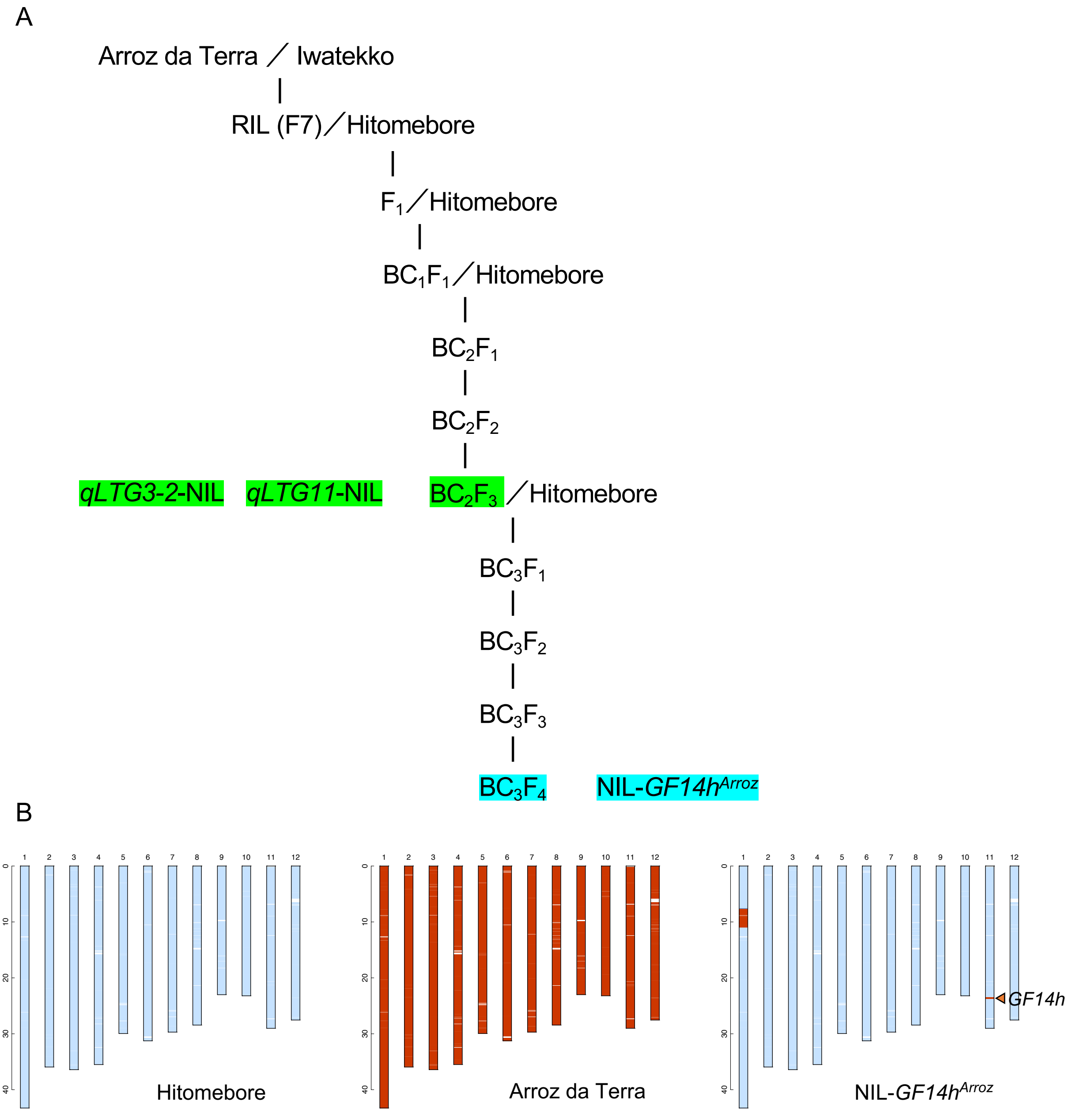

S3 Fig. Generation of a near-isogenic line with high LTG in the Hitomebore background.

(A) Strategy for the development of *qLTG3-2*-NIL, *qLTG11*-NIL, and NIL-$GF14h^{Arroz}$. Molecular markers were used for foreground and background selection. **(B)** Diagram showing the genotype of NIL-$GF14h^{Arroz}$. NIL-$GF14h^{Arroz}$ contains a 172-kb region on chromosome 11 harboring the Arroz da Terra allele of *GF14h*. Light blue bars indicate genomic fragments from Hitomebore; red bars indicate genomic fragments from Arroz da Terra.
