# Supplementary material for: Impact of rice GENERAL REGULATORY FACTOR14h (GF14h) on low-temperature seed germination and its application to breeding
Source: PLoS Genet. 2024 Aug 7;20(8):e1011369. doi: 10.1371/journal.pgen.1011369 (PMC11343456; doi:10.1371/journal.pgen.1011369)
Supplement: S4 Fig — (A) Diagram showing the genotype of qLTG3-2-NIL containing the Arroz da Terra allele at qLTG3-2 on chromosome 3. Light blue bars indicate genomic fragments from Hitomebore; red bars indicate genomic fragments from Arroz da Terra; dark blue bars indicate heterozygous regions. (B) Germination time courses for seeds of Hitomebore, the qLTG3-2-NIL, and Arroz da Terra at 15°C. Values are means ± SD of biologically independent samples (Hitomebore and NIL n = 10, Arroz da Terra n = 5). (PDF) [file pgen.1011369.s004.pdf]

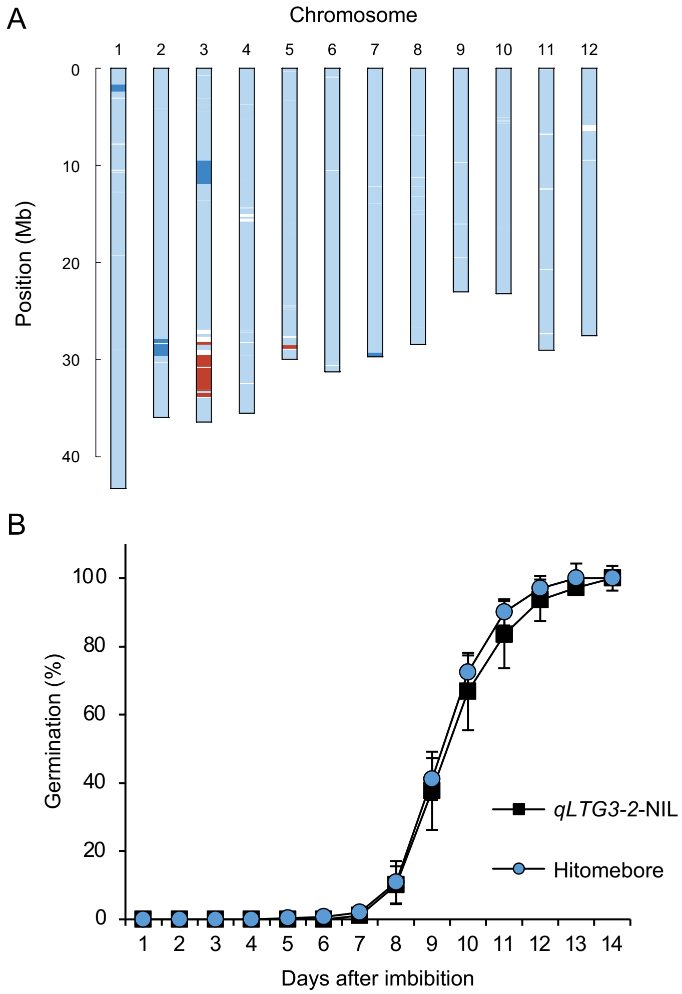

S4 Fig. Summary of *qLTG3-2*.

(A) Diagram showing the genotype of *qLTG3-2*-NIL containing the Arroz da Terra allele at *qLTG3-2* on chromosome 3. Light blue bars indicate genomic fragments from Hitomebore; red bars indicate genomic fragments from Arroz da Terra; dark blue bars indicate heterozygous regions. **(B)** Germination time courses for seeds of Hitomebore and the *qLTG3-2*-NIL at 15° C. Values are means ± SD of biologically independent samples ($n = 10$).
