# Supplementary material for: Impact of rice GENERAL REGULATORY FACTOR14h (GF14h) on low-temperature seed germination and its application to breeding
Source: PLoS Genet. 2024 Aug 7;20(8):e1011369. doi: 10.1371/journal.pgen.1011369 (PMC11343456; doi:10.1371/journal.pgen.1011369)
Supplement: S5 Fig — Germination time courses of seeds from Hitomebore and qLTG11-NIL at 25°C. Values are means ± SD of biologically independent samples (n = 3). Two-tailed t-test was used between qLTG11-NIL and Hitomebore for each time point (*P < 0.05 and **P < 0.01). (PDF) [file pgen.1011369.s005.pdf]

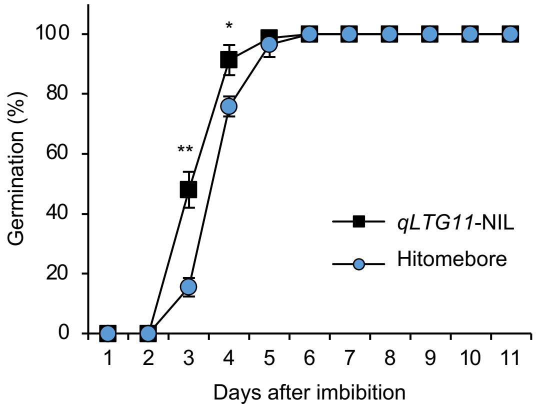

S5 Fig. Seed germination of *qLTG11*-NIL under optimal temperature conditions.

Germination time courses of seeds from Hitomebore and *qLTG11*-NIL at 25° C. Values are means ± SD of biologically independent samples (n = 3). Two-tailed t-test was used between *qLTG11*-NIL and Hitomebore for each time point (*$P < 0.05$ and **$P < 0.01$).
