# Supplementary material for: Impact of rice GENERAL REGULATORY FACTOR14h (GF14h) on low-temperature seed germination and its application to breeding
Source: PLoS Genet. 2024 Aug 7;20(8):e1011369. doi: 10.1371/journal.pgen.1011369 (PMC11343456; doi:10.1371/journal.pgen.1011369)
Supplement: S6 Fig — Dot blot analyses of the genomic sequence in the qLTG11 candidate region between (A) Hitomebore and Nipponbare and (B) Hitomebore and Arroz da Terra, using D-GENIES [62]. Based on the Nipponbare genome (IRGSP-1.0), the genomic region containing the causative gene is located at 23.512–23.564 Mb (approximately 52 kb) on chromosome 11. The genome sequence of Hitomebore is identical to that of Nipponbare. The candidate region corresponds to a fragment of approximately 94 kb in the Arroz da Terra genome. (PDF) [file pgen.1011369.s006.pdf]

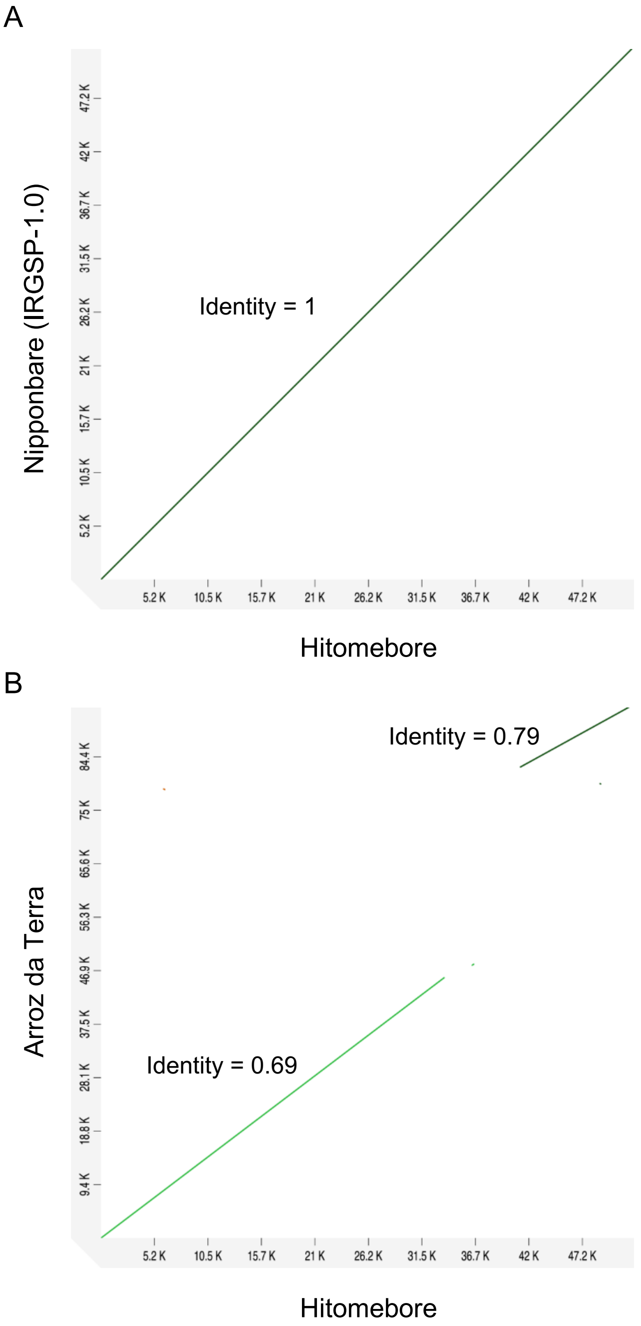


**S6 Fig. Comparison of the *qLTG11* genomic region in Hitomebore, Arroz da Terra, and Nipponbare.**

Dot blot analyses of the genomic sequence in the *qLTG11* candidate region between **(A)** Hitomebore and Nipponbare and **(B)** Hitomebore and Arroz da Terra, using D-GENIES [62]. Based on the Nipponbare genome (IRGSP-1.0), the genomic region containing the causative gene is located at 23.512–23.564 Mb (approximately 52 kb) on chromosome 11. The genome sequence of Hitomebore is identical to that of Nipponbare. The candidate region corresponds to a fragment of approximately 94 kb in the Arroz da Terra genome.
