# Supplementary material for: Impact of rice GENERAL REGULATORY FACTOR14h (GF14h) on low-temperature seed germination and its application to breeding
Source: PLoS Genet. 2024 Aug 7;20(8):e1011369. doi: 10.1371/journal.pgen.1011369 (PMC11343456; doi:10.1371/journal.pgen.1011369)
Supplement: S7 Fig — Total RNA was extracted from Hitomebore and qLTG11-NIL seeds at 0, 1, 2, and 3 days after the onset of seed imbibition under 15 or 25°C temperature conditions, followed by RNA-seq. The sequence reads were mapped to the Nipponbare genome (IRGSP-1.0), and expression data were obtained. (A–B) The expression levels of Os11g0609600 (GF14h) during seed germination under 15°C (A) and 25°C (B) are shown. Data are presented as means ± SE. n = 3 biologically independent samples. (C–D) The expression levels of Os11g0609500 (Jacalin-like lectin domain containing protein) during seed germination under 15°C (C) and 25°C (D) are shown. Data are presented as means ± SE. n = 3 biologically independent samples. (PDF) [file pgen.1011369.s007.pdf]

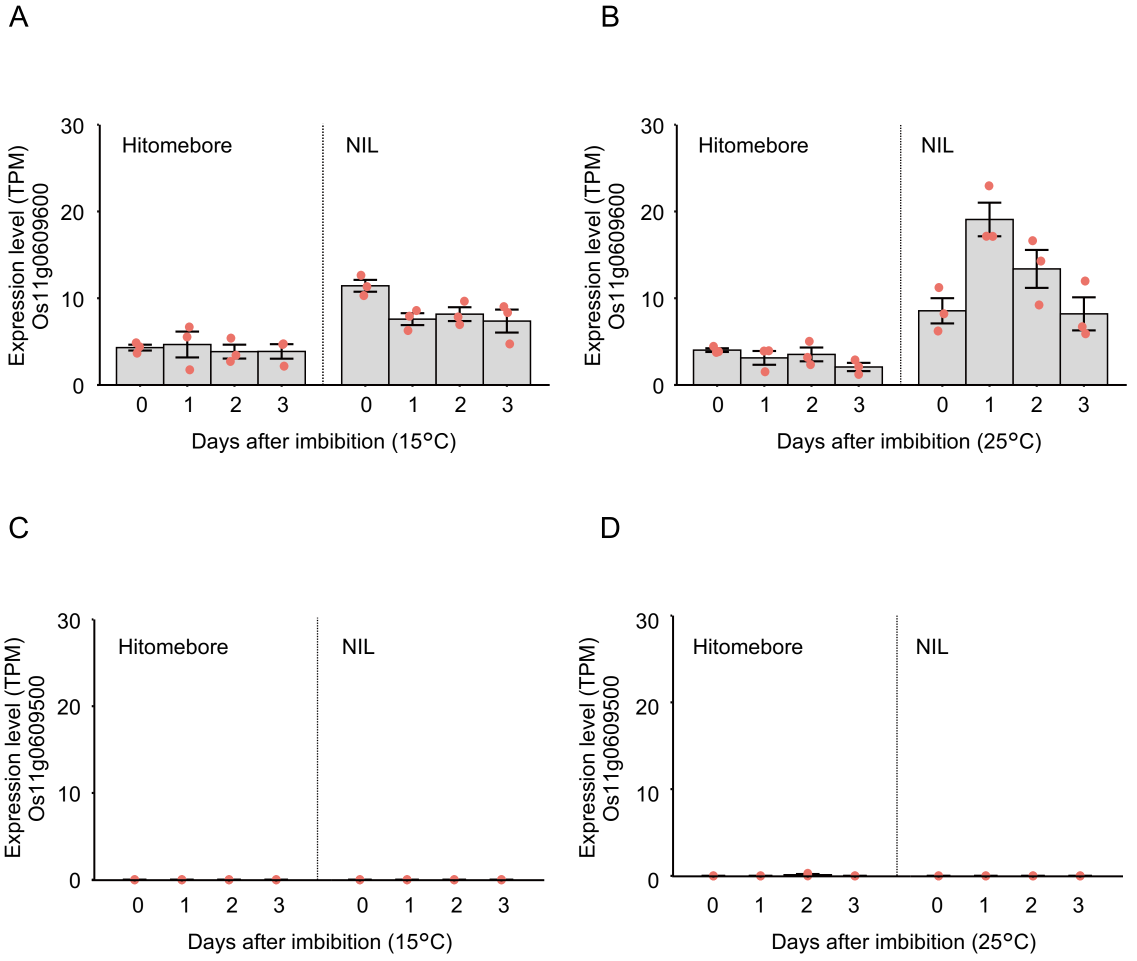

S7 Fig. Expression levels of the two annotated genes in the candidate genomic region of *qLTG11* based on RNA-seq data.

Total RNA was extracted from Hitomebore and *qLTG11*-NIL seeds at 0, 1, 2, and 3 days after the onset of seed imbibition under 15 or 25° C temperature conditions, followed by RNA-seq. The sequence reads were mapped to the Nipponbare genome (IRGSP-1.0), and expression data were obtained. **(A–B)** The expression levels of Os11g0609600 (*GF14h*) during seed germination under 15° C (A) and 25° C (B) are shown. Data are presented as means ± SE. n = 3 biologically independent samples. **(C–D)** The expression levels of Os11g0609500 (*Jacalin-like lectin domain containing protein*) during seed germination under 15° C (C) and 25° C (D) are shown. Data are presented as means ± SE. n = 3 biologically independent samples.
