# Supplementary material for: Impact of rice GENERAL REGULATORY FACTOR14h (GF14h) on low-temperature seed germination and its application to breeding
Source: PLoS Genet. 2024 Aug 7;20(8):e1011369. doi: 10.1371/journal.pgen.1011369 (PMC11343456; doi:10.1371/journal.pgen.1011369)
Supplement: S8 Fig — Arroz da Terra carries a functional GF14h variant. Hitomebore and Nipponbare harbors a loss-of-function variant of GF14h due to a 4-bp deletion (black line). (PDF) [file pgen.1011369.s008.pdf]

```
                       10        20        30        40        50        60
Arroz da Terra  ATGAAGGAGAGGGAGAAGGTGGTGCGCCTGGCCAAGCTCGCCGAGCAGGCGGAGAGATAC
Hitomebore      ATGAAGGAGAGGGAGAAGGTGGTGCGACTGGCCAAGCTCGCCGAGCAGGCGGAGAGATAC
Nipponbare      ATGAAGGAGAGGGAGAAGGTGGTGCGACTGGCCAAGCTCGCCGAGCAGGCGGAGAGATAC

                       70        80        90       100       110       120
Arroz da Terra  GATGATATGGTGGAATTTATGAAAACACTTGCTAGGATGGATGTGGATATGAGTGCTGAA
Hitomebore      GACGATATGGTGGAATTTATGAAGACACTTGCTAGGATGGATGTGGATATGAGTGCTGAA
Nipponbare      GACGATATGGTGGAATTTATGAAGACACTTGCTAGGATGGATGTGGATATGAGTGCTGAA

                      130       140       150       160       170       180
Arroz da Terra  GAGAGGCTTTTATTTTCAGTTGGTTTTAAGAAAACTATTGGTGCCAGGAGAGCATCATGG
Hitomebore      GAGAGGCTTTTATTTTCAGTTGGTTTTAAGAAAACTATTGGTGCCAGGAGAGCATCATGG
Nipponbare      GAGAGGCTTTTATTTTCAGTTGGTTTTAAGAAAACTATTGGTGCCAGGAGAGCATCATGG

                      190       200       210       220       230       240
Arroz da Terra  AGAATCCTTGAATCACTCGAGCAGAAGGTGACAGCTGGTGAGCAACCAGGTGTAACAATA
Hitomebore      AGAATCCTTGAATCACTTGAGCAGAAGGTGACAGCTGGTGACCAACCAGGTGTAACAATA
Nipponbare      AGAATCCTTGAATCACTTGAGCAGAAGGTGACAGCTGGTGACCAACCAGGTGTAACAATA

                      250       260       270       280       290       300
Arroz da Terra  AATGGCTACAAAAAGAAAGTAGAGGATGAACTAAGGGCAGTTTGCAATGAAGTATTATCA
Hitomebore      AATGGCTACAAAAAGAAAGTAGAGGATGAACTAAGGGCAGTTTGCAATGAAGTATTATCA
Nipponbare      AATGGCTACAAAAAGAAAGTAGAGGATGAACTAAGGGCAGTTTGCAATGAAGTATTATCA

                      310       320       330       340       350       360
Arroz da Terra  ATTATTGCTATTCATTGCCTTCCATTGGCCAATTCGGGTGAAAATGTCGTGTTCTTTTAT
Hitomebore      ATTATTGCTATTCATTGCCTTCCATTGGCCAATTCGGGTGAAAATGTCGTGTTCTTTTAT
Nipponbare      ATTATTGCTATTCATTGCCTTCCATTGGCCAATTCGGGTGAAAATGTCGTGTTCTTTTAT

                      370       380       390       400       410       420
Arroz da Terra  AAGATGAAGGGTGATTACTACCGTTACTTGGCTGAATTTAGCACTGGAACTGAAAAGAAG
Hitomebore      AAGATGAAGGGTGACTACTACCGTTACTTGGCTGAATTTAGCACTGGAACTGAAAAGAAG
Nipponbare      AAGATGAAGGGTGACTACTACCGTTACTTGGCTGAATTTAGCACTGGAACTGAAAAGAAG

                      430       440       450       460       470       480
Arroz da Terra  GCTGCTACTGATCAGTCCCTTATGGCCTATCAGCACGCCATGGTTGTTTCCTCCAGTGAG
Hitomebore      GCTGCTACTGATCAGTCCCTTATGGCCTATCAGCACGCCATGGTTGTTGCCTCCAGTGAG
Nipponbare      GCTGCTACTGATCAGTCCCTTATGGCCTATCAGCACGCCATGGTTGTTGCCTCCAGTGAG

                      490       500       510       520       530       540
Arroz da Terra  CTTTCTCCTGTCCATCAATTCAGGCTTGGCCTTGCGCTCAACTTTTCAGTCTTCTTTTAT
Hitomebore      CTTTCTCCTGCCCATCAATTCAGGCTTGGCCTTGCGCTCAACTTTTCAGTCTTCTT----
Nipponbare      CTTTCTCCTGCCCATCAATTCAGGCTTGGCCTTGCGCTCAACTTTTCAGTCTTCTT----

                      550       560       570       580       590       600
Arroz da Terra  GAGATAATGAACTCTCCTGAGAGAGCTAGCCAAGTGGCAAAACAAGCACTTGATGAGGCT
Hitomebore      GAGATAA-----------------------------------------------------
Nipponbare      GAGATAA-----------------------------------------------------

                      610       620       630       640       650       660
Arroz da Terra  ACTGCTGAGATTAATTCTGCTGGTGTGGAGGGTTACAAGGACAGCATGCTTATGATGCAG
Hitomebore      ------------------------------------------------------------
Nipponbare      ------------------------------------------------------------

                      670       680       690       700       710       720
Arroz da Terra  CTCCTGAAGGAGAACCTGGCATTGTGGACATCAGAACTAACTGGAGGTGAAACATCTAAG
Hitomebore      ------------------------------------------------------------
Nipponbare      ------------------------------------------------------------

                      730       740
Arroz da Terra  GACGATGACGTTGTTATGGAGGGCTAA
Hitomebore      ---------------------------
Nipponbare      ---------------------------
```

S8 Fig. Multiple DNA sequence alignment of *GF14h* variants.

Arroz da Terra carries a functional *GF14h* variant. Hitomebore and Nipponbare harbors a loss-of-function variant of *GF14h* due to a 4-bp deletion (black line).
