# Supplementary material for: Impact of rice GENERAL REGULATORY FACTOR14h (GF14h) on low-temperature seed germination and its application to breeding
Source: PLoS Genet. 2024 Aug 7;20(8):e1011369. doi: 10.1371/journal.pgen.1011369 (PMC11343456; doi:10.1371/journal.pgen.1011369)
Supplement: S9 Fig — Top, diagram showing the GF14h locus, with the locations of the two sgRNA target sites marked by inverted red triangles. Bottom, sequencing results of putative gf14h mutants. The sgRNA target sites are underlined, and the PAMs are highlighted. The mutation sites in GF14hArroz for the four mutants (gf14h-1, gf14h-2, gf14h-3, and gf14h-4) are indicated. (PDF) [file pgen.1011369.s009.pdf]

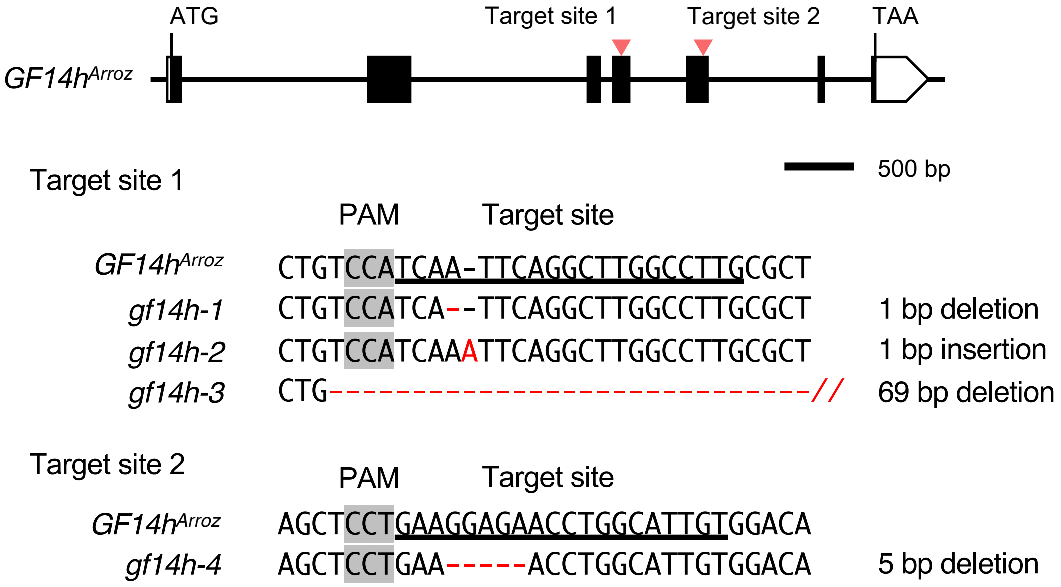

S9 Fig. CRISPR/Cas9-mediated genome editing of *GF14h*.

Top, diagram showing the *GF14h* locus, with the locations of the two sgRNA target sites marked by inverted red triangles. Bottom, sequencing results of putative *gf14h* mutants. The sgRNA target sites are underlined, and the PAMs are highlighted. The mutation sites in *GF14h*^{Arroz} for the four mutants (*gf14h-1*, *gf14h-2*, *gf14h-3,* and *gf14h-4*) are indicated.
