# Supplementary material for: Impact of rice GENERAL REGULATORY FACTOR14h (GF14h) on low-temperature seed germination and its application to breeding
Source: PLoS Genet. 2024 Aug 7;20(8):e1011369. doi: 10.1371/journal.pgen.1011369 (PMC11343456; doi:10.1371/journal.pgen.1011369)
Supplement: S10 Fig — (A) Representative photographs showing seed germination in wild-type harboring Arroz-type GF14h (WTArroz) and CRISPR/Cas9 knockout lines (gf14-1) at 3 days after the onset of seed imbibition. Scale bar, 1 cm. (B) Seed germination rate of WTArroz and its CRISPR/Cas9 knockout lines at 2 days of seed imbibition at 25°C. The two target constructs (S9 Fig) were introduced into the qLTG11-NIL line. Data are means ± standard error (WTArroz, gf14h-1, gf14h-2 and gf14h-4, n = 3; gf14h-3, n = 2). Different lowercase letters indicate significant differences based on Tukey’s HSD test (P < 0.05). (PDF) [file pgen.1011369.s010.pdf]

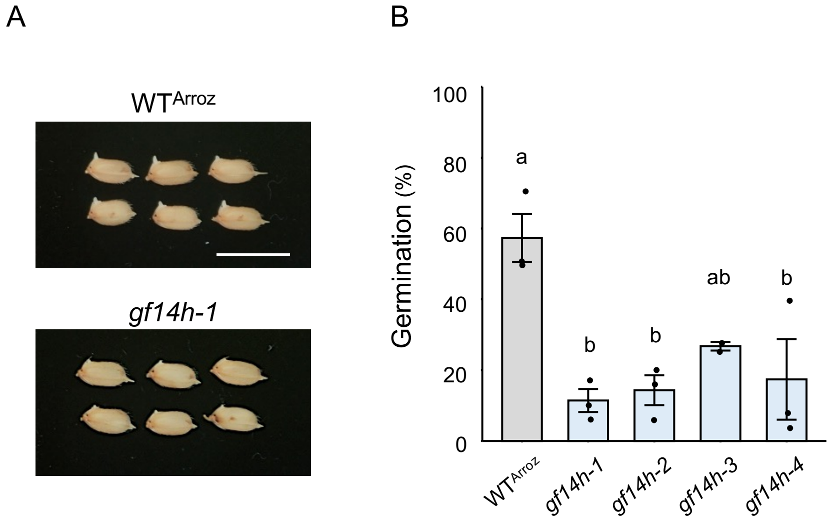

S10 Fig. Effect of *GF14h* mutation on optimal-temperature germination.
(A) Representative photographs showing seed germination in wild-type harboring Arroz-type *GF14h* (WT^{Arroz}) and CRISPR/Cas9 knockout lines (*gf14-1*) at 3 days after the onset of seed imbibition. Scale bar, 1 cm. **(B)** Seed germination rate of WT^{Arroz} and its CRISPR/Cas9 knockout lines at 2 days of seed imbibition at 25° C. The two target constructs (S9 Fig) were introduced into the *qLTG11*-NIL line. Data are means ± standard error (WT^{Arroz}, *gf14h-1*, *gf14h-2* and *gf14h-4*, n = 3; *gf14h-3*, n = 2). Different lowercase letters indicate significant differences based on Tukey's HSD test ($P < 0.05$).
