# Supplementary material for: Impact of rice GENERAL REGULATORY FACTOR14h (GF14h) on low-temperature seed germination and its application to breeding
Source: PLoS Genet. 2024 Aug 7;20(8):e1011369. doi: 10.1371/journal.pgen.1011369 (PMC11343456; doi:10.1371/journal.pgen.1011369)
Supplement: S11 Fig — The GF14hArroz overexpression construct was introduced into Hitomebore. OsActin1 (Os03g0718100) was used for normalization. Values are means ± SE (n = 3 or 4). Different lowercase letters indicate significant differences based on Tukey’s HSD test (P < 0.001). (PDF) [file pgen.1011369.s011.pdf]

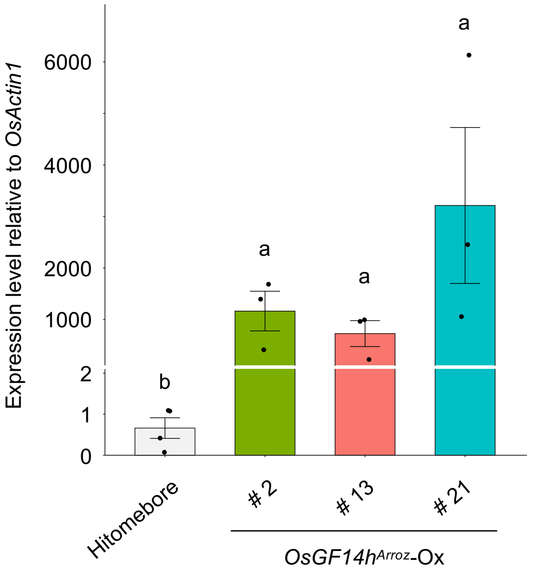

S11 Fig. Relative *GF14h* expression levels in germinating seeds of *GF14h*^{Arroz} overexpression lines and the parental line.

The *GF14h*^{Arroz} overexpression construct was introduced into Hitomebore. *OsActin1* (Os03g0718100) was used for normalization. Values are means ± SE (*n* = 3 or 4). Different lowercase letters indicate significant differences based on Tukey's HSD test ($P < 0.001$).
