# Supplementary material for: Impact of rice GENERAL REGULATORY FACTOR14h (GF14h) on low-temperature seed germination and its application to breeding
Source: PLoS Genet. 2024 Aug 7;20(8):e1011369. doi: 10.1371/journal.pgen.1011369 (PMC11343456; doi:10.1371/journal.pgen.1011369)
Supplement: S12 Fig — The GF14h genomic sequences obtained from 411 O. sativa varieties and 11 O. rufipogon accessions were used for analysis (S1 Table). The haplotype network was reconstructed by the median joining network algorithm [60] implemented in Popart v1.7 [61]. The haplotype Hap1 evolved from Hap2 by acquiring the 4-bp sequence, resulting in a nonfunctional GF14h gene. The Hitomebore cultivar contains Hap1 (nonfunctional), and the Arroz da Terra cultivar contains Hap9 (functional). (PDF) [file pgen.1011369.s012.pdf]

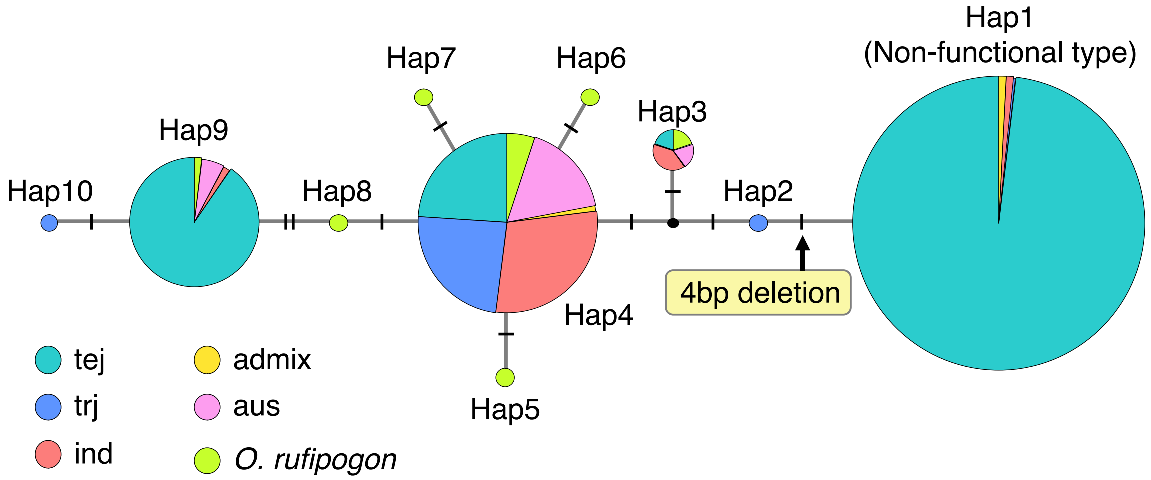

S12 Fig. Haplotype network of *GF14h*.
The *GF14h* genomic sequences obtained from 411 *O. sativa* varieties and 11 *O. rufipogon* accessions were used for analysis (S1 Table). The haplotype network was reconstructed by the median joining network algorithm [60] implemented in Popart v1.7 [61]. The haplotype Hap1 evolved from Hap2 by acquiring the 4-bp sequence, resulting in a nonfunctional *GF14h* gene. The Hitomebore cultivar contains Hap1 (nonfunctional), and the Arroz da Terra cultivar contains Hap9 (functional).
