# Supplementary material for: Impact of rice GENERAL REGULATORY FACTOR14h (GF14h) on low-temperature seed germination and its application to breeding
Source: PLoS Genet. 2024 Aug 7;20(8):e1011369. doi: 10.1371/journal.pgen.1011369 (PMC11343456; doi:10.1371/journal.pgen.1011369)
Supplement: S13 Fig — Germination time courses of seeds from Hitomebore (blue circles) and the NIL-GF14hArroz (pink triangles) under wet conditions at 28°C. Seeds were harvested from tagged panicles 30 days after heading. Values are means ± SE of biologically independent samples (n = 3). The P-values calculated from t-tests at each time point are shown in the figure. (PDF) [file pgen.1011369.s013.pdf]

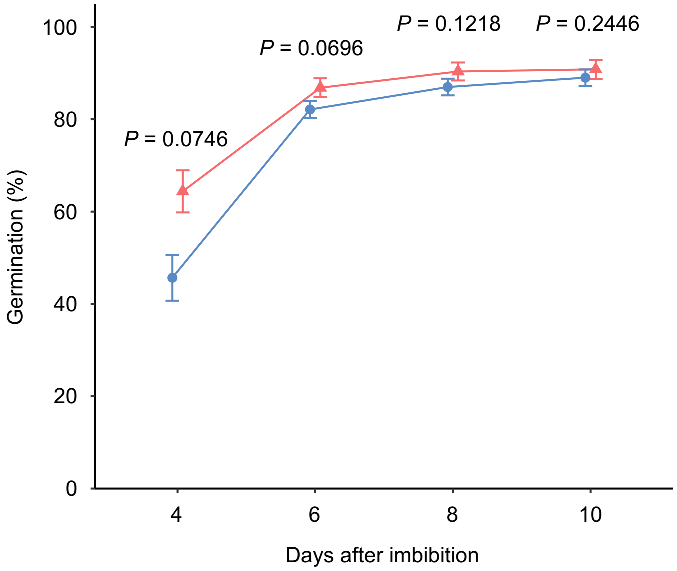

S13 Fig. Pre-harvest sprouting of NIL-*GF14h*^{Arroz}. Germination time courses of seeds from Hitomebore (blue circles) and the NIL-*GF14h*^{Arroz} (pink triangles) under wet conditions at 28° C. Seeds were harvested from tagged panicles 30 days after heading. Values are means ± SE of biologically independent samples (n = 3). The P-values calculated from t-tests at each time point are shown in the figure.
