# Supplementary material for: Impact of rice GENERAL REGULATORY FACTOR14h (GF14h) on low-temperature seed germination and its application to breeding
Source: PLoS Genet. 2024 Aug 7;20(8):e1011369. doi: 10.1371/journal.pgen.1011369 (PMC11343456; doi:10.1371/journal.pgen.1011369)
Supplement: S14 Fig — (A) Diagram of the sequence around the 4-bp InDel of GF14h. In the Hitomebore (loss-of-function) allele, the 4-bp deletion creates a SmlI restriction site. (B) Genotyping of the 4-bp deletion in GF14h. A genomic fragment containing the 4-bp InDel of GF14h was amplified by PCR and digested with SmlI. The products were separated on a 3% (w/v) agarose gel and stained with Midori Green. The PCR product from GF14hArroz (approximately 500 bp) was not cleaved, whereas the PCR product from GF14hHitomebore was cleaved, producing two fragments of approximately 250 bp each. Both bands were detected in heterozygous plants. (PDF) [file pgen.1011369.s014.pdf]

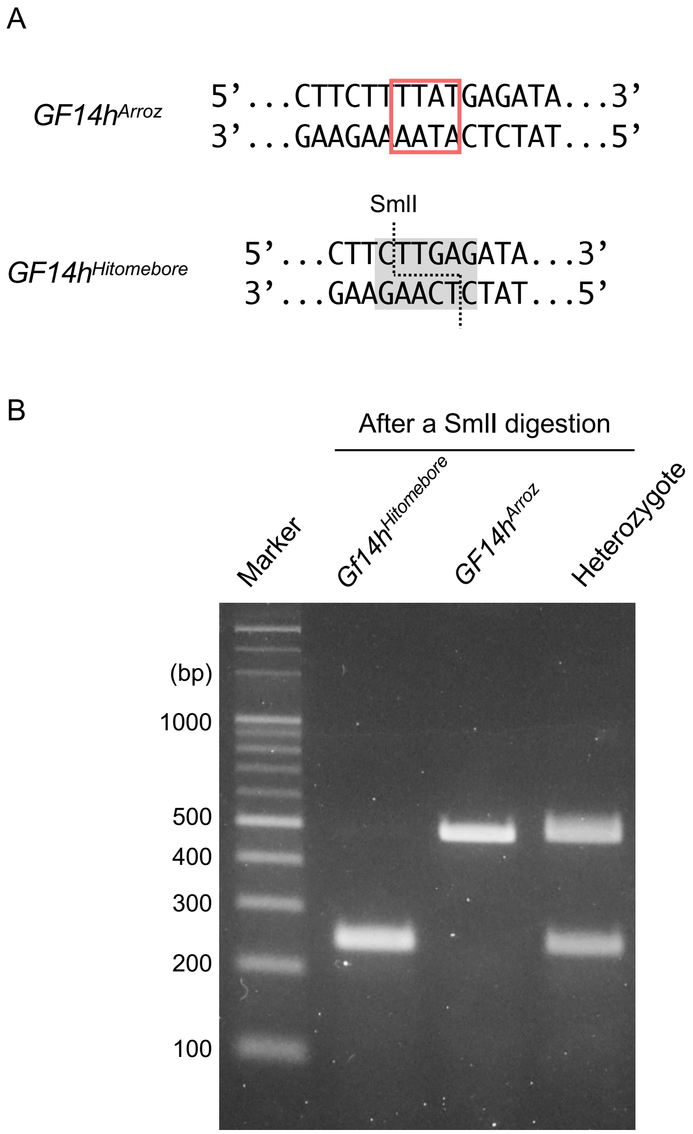

S14 Fig. Development of a functional marker based on the 4-bp deletion in *GF14h*.

(A) Diagram of the sequence around the 4-bp InDel of *GF14h*. In the Hitomebore (loss-of-function) allele, the 4-bp deletion creates a SmlI restriction site. **(B)** Genotyping of the 4-bp deletion in *GF14h*. A genomic fragment containing the 4-bp InDel of *GF14h* was amplified by PCR and digested with SmlI. The products were separated on a 3% (w/v) agarose gel and stained with Midori Green. The PCR product from *GF14h*^{Arroz} (approximately 500 bp) was not cleaved, whereas the PCR product from *GF14h*^{Hitomebore} was cleaved, producing two fragments of approximately 250 bp each. Both bands were detected in heterozygous plants.
