# Supplementary material for: Impact of rice GENERAL REGULATORY FACTOR14h (GF14h) on low-temperature seed germination and its application to breeding
Source: PLoS Genet. 2024 Aug 7;20(8):e1011369. doi: 10.1371/journal.pgen.1011369 (PMC11343456; doi:10.1371/journal.pgen.1011369)
Supplement: Attachment — Submitted filename: Review on Sugimura et al.docx [file pgen.1011369.s021.docx]

Review on Sugimura et al.,

The manuscript by Sugimura et al. investigates the seed germination at low temperature (15°C) in rice using a previously generated set of 200 RILs between two rice varieties (Arroz da Terra and Iwatekko). The germination of rice seeds at low temperature is an agrotechnical bottleneck and therefore an important trait. Applying the QTL-seq pipeline established previously in the lab, the authors identified three QTLs, including a known QTL and two new ones. One of the new QTLs showed no effect in the generated NILs while the other contained the gene GF14h, a 14-3-3 regulatory protein. Using knock-out and overexpressing lines the authors showed the role of GF14h to promote germination at low temperature. The authors also analyzed the presence of functional alleles of these genes in the available rice varieties and landraces. The generation of a NIL line harboring the functional GF14h allele in an elite cultivar background, which has non-functional endogenous allele would be a potential interest of rice breeders. I note that the two new genome assembly (Hitomebore and Arroz da Terra) based on long reads is valuable information, too.

The manuscript is a solid work with new data and an interesting conclusion that genetic breeding in the past was strongly selective to lower pre-harvest sprouting that possibly resulted the low germination ability at lower (non-optimal) temperatures as a trade-off.

While the manuscript is overall well written and depicts the importance of the study, some data are not shown or not presented well.

I suggest major revision considering my main comments, mainly regarding data not shown properly.

Major comments:

- 1., The Fig. 1A is not sufficient to describe the fundamental phenotype. A germination-rate plot is needed, for example comparing all the used varieties at optimal and suboptimal temperature over time. Particularly, as similar setup was used for the QTL phenotyping, that is not shown too. While on later figures the authors use more precise numbers, in the first part of the manuscript the phenotype description is very vague, for example (line 116) “germinates more vigorously” without mentioning the exact quantification or numbers. For scientific correctness I suggest adding exact values, for example the time point when the germination reached 50% (T50) or as on Supplementary Fig. 1; the germination after 8-9 days. Determining the area under the line (integration) of germination rate per time might be informative, too. Statistical tests are also needed to show at which time point do they differ in the germination. This applies also on Fig. 1D.
- 2., Similarly, the germination data on the RILs on Supplementary Figure 1 should be shown in more detail. At least, the selected 20-20 RIL lines must be shown individually at one or more timepoint.
- 3. The authors perform an RNAseq experiment, but they literally show none of the data (line 162-165). This must be presented or left out from the manuscript. How much does the GF14h express in the two lines? How much does the other gene (Os11g0609500) express? Also, the RNAseq data matrix should be added as a supplementary file with gene IDs and RPKM (or equivalent) values. I cannot find the BioProject accession, mentioned on line451.
- 4. Following on the previous comment, there are no data shown on the other gene of the qLTG11 region (Os11g069500). While the data on GF14h are convincing, I`m wondering if the authors generated knock-out or overexpressing line with the other gene. If this gene is not expressing at seed stage, the data must be shown.

Minor comments:

- Line 36: Without expert`s insight, it is not clear in the Abstract what “high-LTG” (reads as high-low-temperature germination) means here. I recommend writing it on an easier way in the abstract, as: …from rice variety Arroz da Terra, germinating at lower temperature, using QTL-seq.
- Line 121: the exact condition of phenotyping must be added in the text and not just in the materials and methods. For the phenotyping condition, was the temperature set to 13 or 15°C? I see different numbers in the Materials and Methods and on Supplementary Figure 1 description.
- Line140: something is missing from this sentence; ‘We detected no clear effect’ of what?
- Line 141: instead of “germinability was more vigorous” I suggest describing the exact quantified results.
- Line 141: the chromosome map of qLTG11-NIL is missing.
- Line 143, related to Supplementary Figure 5: statistical test is needed.
- Line 164: expressional data need to be shown side-by-side for both genes in the 52 kb region. Is the other gene (Os11g0609500) expressed in seeds?
- Line 167: is the other gene sequence identical? The Authors never mention the other gene again.
- Line 193: ‘LTG rates’. It is not a rate.
- Line 217: The next most common where/among which one? I assume in land races. Please clarify the sentence.
- Line 237: Although I understand the authors, I am not sure that it is correct to write `slightly more susceptible`if the p-value is >0.07. As I found it an important piece of data, I recommend to insert on the main figure.
- Fig 1D: statistical test is needed.
- Fig. 2D: do these data refer to a RT-qPCR? It should be clearly described in the legends.
- Fig4: it would be interesting to see the germination properties of some of the varieties which have both functional alleles, for example Hokuriku193.
