# Supplementary material for: Impact of rice GENERAL REGULATORY FACTOR14h (GF14h) on low-temperature seed germination and its application to breeding
Source: PLoS Genet. 2024 Aug 7;20(8):e1011369. doi: 10.1371/journal.pgen.1011369 (PMC11343456; doi:10.1371/journal.pgen.1011369)
Supplement: Attachment — Submitted filename: Response_to_reviewers.docx [file pgen.1011369.s022.docx]

**Editor comments**

Thank you very much for submitting your Research Article entitled 'Impact of rice *GENERAL REGULATORY FACTOR14h* (*GF14h*) on low-temperature seed germination and its application to breeding' to PLOS Genetics.

First, apologies for the unusually long processing period. We have tried hard to obtain a third independent review, unfortunately without success. However, as both submitted reviews appreciate the importance of the topic but recommend a substantial revision of the manuscript, we decided to make their comments available to you now, so that you can consider them in a revised version. Based on the reviews, we will not be able to accept this version of the manuscript, but we would be willing to review a much-revised version. We cannot, of course, promise publication at that time.

As you will see from the reviewer’s comments, they found discrepancies between experimental details, incomplete data sets, and vague statements when precise quantification is needed. Differences in germination behavior at certain conditions should consider differences other than only the modified parameter, and there are more complex ways to characterize germination kinetics. In addition, if referring to transcriptome data, these need to be made available, and expression data should be quantitative. Please discuss with your co-authors if you can give more information on the other potentially relevant genes, or on other regulatory components than ABA and GA. We hope that these comments will help to prepare a substantially revised version.

Thank you very much for your evaluation and comments. We have revised the manuscript by integrating feedback from the coworkers and addressing the points suggested by the reviewers.

We sincerely hope that the revised manuscript is now acceptable for publication in PLOS Genetics.

**Point-by-point reply to the review comments**

**Reviewer #1**: Direct seeding of rice is becoming popular in recent years. However, this approach requires varieties with vigorous low temperature germination especially when sown under cold conditions in the early spring. This manuscript revealed the candidate gene *GF14h* regulates low temperature germination in rice. The authors also investigated the distribution of functional *GF14h* allele in the popular varieties during modern breeding. Further, the contribution of functional *GF14h* allele from Arroz da Terra was confirmed for rice direct seeding in cold regions. Several comments are listed below.

Thank you very much for your positive evaluation and comments. We have revised our manuscript by addressing the points suggested by you.

1. It has been revealed that GF14h regulates rice seed germination through abscisic acid and gibberellin signaling pathways. In this study, the mechanism of GF14h influencing low temperature germination was still unclear. Whether other novel factors, except abscisic acid and gibberellin signaling, involved in the GF14h medicated regulation of low temperature germination?

Thank you for your comment. However, it is difficult to infer molecular mechanisms involved in LTG beyond those reported by previous papers with the data available in the current study. We would like to consider it in the future study.

2. The germination rates of RIL population were determined at 8 days of seed imbibition at 13°C. But 15°C was used for NIL, knockout and overexpression lines. Why?

Thank you for your comment. While the LTG test for QTL-seq was performed at 13°C (Takagi et al. 2013 Plant J.) which is a severe condition for rice plant, subsequent analyses using NIL, knockout lines, and overexpression lines were performed at 15°C for two reasons: 1) The major LTG factor qLTG3-1 analysis had been reported to have been tested at 15°C (Fujino et al. 2008 PNAS); 2) By focusing on only one of the three QTLs, we considered the condition at 13°C might make it difficult to evaluate the effect of a single QTL on LTG performance.

We have consistently performed germination tests at 15°C, except for the QTL-seq analysis. So, we believe that there is no inconsistency in the conclusion that GF14h is responsible for LTG.

We have revised the text as follows.

Line 128 in the revised manuscript:

We phenotyped all RILs for LTG at 13ºC and …

Lines 384-385 in the revised manuscript:

For QTL-seq analysis, germination tests were conducted at 13°C [31].

3. Results showed that *qLTG11*-NIL seeds also germinated more rapidly than Hitomebore seeds under normal conditions (25ºC). For other knockout and overexpression lines, how about the germination phenotype under normal conditions (25ºC)? The difference of germination speed will influence the low temperature germination?

Thank you for your comment. To answer the reviewer's point, we performed a germination test at 25°C using the knockout lines for which we had sufficient number of seeds. The results indicated that the germination rate was generally lower in the *GF14h* knockout lines compared to WT^Arroz^. This finding aligns with previous reports that *GF14h* promotes seed germination at 30°C (Sun et al., 2022). This result has been included in S10 Fig and described in the text (lines 205-207).

Lines 205-207 in the revised manuscript:

We also found that the knockout lines tended to have lower germination rates than WT^Arroz^ under normal temperature conditions (25°C) (S10 Fig), which is consistent with the previous report [27].

4. NIL-*GF14h^Arroz^* was slightly more susceptible to pre-harvest sprouting than Hitomebore. It is also a challenge for the future rice breeding.

Thank you for your constructive feedback. Should NIL-*GF14h^Arroz^* encounter challenges with pre-harvest sprouting at the practical level, we will consider introducing QTLs associated with pre-harvest sprouting resistance, such as *Seed Dormancy 4* (Sugimoto et al. 2010 PNAS). We added this consideration to the discussion (Lines 354-357).

Lines 354-357 in the revised manuscript:

…On the other hand, enhancing LTG may conversely increase the risk of pre-harvest sprouting. Should the level of pre-harvest sprouting in NIL-*GF14h^Arroz^* pose practical issues, the pyramiding of QTLs for pre-harvest sprouting resistance, such as *Seed Dormancy 4* [39], may offer a solution.

5. Whether *GF14h* regulates the seedling establishment or seedling growth at low temperatures?

Thank you for your question. The answer to the question is currently unknown,　but as mentioned in lines 347-349 in the revised manuscript, clarifying the involvement of *GF14h* in seedling establishment and seedling growth under low-temperature conditions will be an important issue to address.

Lines 347-349 in the revised manuscript

However, whether NIL-*GF14h^Arroz^* exhibits strong seedling vigor at low temperatures in rice fields remains to be determined.

**Reviewer #2**:

Review on Sugimura et al.,

The manuscript by Sugimura et al. investigates the seed germination at low temperature (15°C) in rice using a previously generated set of 200 RILs between two rice varieties (Arroz da Terra and Iwatekko). The germination of rice seeds at low temperature is an agrotechnical bottleneck and therefore an important trait. Applying the QTL-seq pipeline established previously in the lab, the authors identified three QTLs, including a known QTL and two new ones. One of the new QTLs showed no effect in the generated NILs while the other contained the gene GF14h, a 14-3-3 regulatory protein. Using knock-out and overexpressing lines the authors showed the role of GF14h to promote germination at low temperature. The authors also analyzed the presence of functional alleles of these genes in the available rice varieties and landraces. The generation of a NIL line harboring the functional GF14h allele in an elite cultivar background, which has non-functional endogenous allele would be a potential interest of rice breeders. I note that the two new genome assembly (Hitomebore and Arroz da Terra) based on long reads is valuable information, too.

The manuscript is a solid work with new data and an interesting conclusion that genetic breeding in the past was strongly selective to lower pre-harvest sprouting that possibly resulted the low germination ability at lower (non-optimal) temperatures as a trade-off.

While the manuscript is overall well written and depicts the importance of the study, some data are not shown or not presented well.

I suggest major revision considering my main comments, mainly regarding data not shown properly.

Thank you very much for your positive evaluation and the comments. We have revised our manuscript by addressing the points suggested by you.

Major comments:

1., The Fig. 1A is not sufficient to describe the fundamental phenotype. A germination-rate plot is needed, for example comparing all the used varieties at optimal and suboptimal temperature over time. Particularly, as similar setup was used for the QTL phenotyping, that is not shown too. While on later figures the authors use more precise numbers, in the first part of the manuscript the phenotype description is very vague, for example (line 116) “germinates more vigorously” without mentioning the exact quantification or numbers. For scientific correctness I suggest adding exact values, for example the time point when the germination reached 50% (T50) or as on Supplementary Fig. 1; the germination after 8-9 days. Determining the area under the line (integration) of germination rate per time might be informative, too. Statistical tests are also needed to show at which time point do they differ in the germination. This applies also on Fig. 1D.

Thank you for your valuable advice. We presented the seed germination rate plots for Arroz da Terra, Iwatekko, and Hitomebore under both optimal and suboptimal conditions (Fig 1B and 1C). The obtained data were analyzed statistically, describing the quantitative results (Lines 116-125). Additionally, the photographs of germinating seeds were updated (Fig 1A). In Fig 1D, we illustrated the germination plots for qLTG11-NIL and Hitomebore and provided the corresponding quantitative results (Lines 149-152).

Lines 116-125 in the revised manuscript:

We investigated seed germination characteristics of a Portuguese rice variety Arroz da Terra and a Japanese varieties Iwatekko and Hitomebore. Under low-temperature conditions (15ºC), Arroz da Terra exhibited significantly higher germination rates compared to Iwatekko and Hitomebore during days 7−15, particularly showing a 30−40% increase in germination rate 10−11 days after imbibition (Fig 1A and 1B). Similarly, at normal temperature conditions (25ºC), Arroz da Terra showed superior germination rates 2−4 days after imbibition, especially with a 30% difference observed 3 days after imbibition (Fig 1A and 1C). These findings indicate that Arroz da Terra exhibits more vigorous germination under normal and low-temperature conditions than the Japanese cultivars Iwatekko and Hitomebore.

Lines 149-152 in the revised manuscript:

By contrast, *qLTG11*-NIL showed a significantly higher rate of germination than Hitomebore 7−12 days after imbibition at 15ºC, particularly after 8 and 9 days, showing a difference of more than 40% (Fig 1E and 1F), indicating that *qLTG11* enhances LTG.

2., Similarly, the germination data on the RILs on Supplementary Figure 1 should be shown in more detail. At least, the selected 20-20 RIL lines must be shown individually at one or more timepoint.

Thank you for your comment. We have added the germination rate data of the 20-20 RILs selected for QTL-seq in Supplementary Figures S1B and S1C.

3. The authors perform an RNAseq experiment, but they literally show none of the data (line 162-165). This must be presented or left out from the manuscript. How much does the GF14h express in the two lines? How much does the other gene (Os11g0609500) express? Also, the RNAseq data matrix should be added as a supplementary file with gene IDs and RPKM (or equivalent) values. I cannot find the BioProject accession, mentioned on line451.

Thank you for your helpful advice. We have created a graph illustrating the expression levels of Os11g0609600 (*GF14h*) and Os11g0609500 during seed germination (as shown in S7 Fig), which indicates that *GF14h* is expressed in seeds while Os11g0609500 is not. This information has been described in the text (lines174-176). In addition, we have included RNA-seq expression matrix data (S1 Table).

BioProject accession is now available to the public, and we have created a supplementary table (S5 Table) with information on SRA number, etc.

Lines174-176 in the revised manuscript:

…and *qLTG11*-NIL, whereas Os11g0609500 (*Jacalin-like lectin domain containing protein*) was not expressed in seeds (S7 Fig), thus suggesting that *GF14h* is a strong candidate gene for LTG. The GF14h …

4. Following on the previous comment, there are no data shown on the other gene of the *qLTG11* region (Os11g069500). While the data on *GF14h* are convincing, I`m wondering if the authors generated knock-out or overexpressing line with the other gene. If this gene is not expressing at seed stage, the data must be shown.

Thank you for your comment. As mentioned earlier, our RNA-seq analysis (see S7 Fig) revealed that Os11g0609500 is not expressed in seeds.

Minor comments:

Line 36: Without expert`s insight, it is not clear in the Abstract what “high-LTG” (reads as high-low-temperature germination) means here. I recommend writing it on an easier way in the abstract, as: …from rice variety Arroz da Terra, germinating at lower temperature, using QTL-seq.

Thank you for your valuable advice. To enhance the reader’s comprehension, we have revised the text as follows.

Line 37 in the revised manuscript:

… Arroz da Terra, which shows rapid seed germination at lower temperatures, using QTL-seq.

Line 121: the exact condition of phenotyping must be added in the text and not just in the materials and methods. For the phenotyping condition, was the temperature set to 13 or 15°C? I see different numbers in the Materials and Methods and on Supplementary Figure 1 description.

Thank you for your comment. While the LTG test for QTL-seq was performed at 13°C (Takagi et al. 2013 Plant J.) which is a severe condition for rice plant, subsequent analyses using NIL, knockout lines, and overexpression lines were performed at 15°C for two reasons: 1) The major LTG factor qLTG3-1 analysis had been reported to have been tested at 15°C (Fujino et al. 2008 PNAS); 2) By focusing on only one of the three detected QTLs, we considered the condition at 13°C might make it difficult to evaluate the effect of a single QTL on LTG performance.

We have revised the text as follows.

Line 128 in the revised manuscript:

We phenotyped all RILs for LTG at 13ºC and …

Lines 384-385 in the revised manuscript:

For QTL-seq analysis, germination tests were conducted at 13°C [31].

Line140: something is missing from this sentence; ‘We detected no clear effect’ of what?

Thank you for your comment. The text has been revised.

Line148 in the revised manuscript:

We detected no clear effect of *qLTG3-2* on LTG, as *qLTG3-2*-NIL and Hitomebore showed similar seed germination rates at 15ºC.

Line 141: instead of “germinability was more vigorous” I suggest describing the exact quantified results.

Thank you for your suggestion. We revised the phrasing to the exact quantified results.

Line 149-151 in the revised manuscript:

By contrast, *qLTG11*-NIL showed a significantly higher rate of germination than Hitomebore 7−12 days after imbibition at 15ºC, particularly after 8 and 9 days, showing a difference of more than 40% (Fig 1E and 1F), indicating that *qLTG11* enhances LTG.

Line 141: the chromosome map of qLTG11-NIL is missing.

Thank you for your comment. The chromosome map of *qLTG11*-NIL is shown in Fig 1E.

Line 143, related to Supplementary Figure 5: statistical test is needed.

Thank you for your suggestion. We conducted statistical tests.

Line 164: expressional data need to be shown side-by-side for both genes in the 52 kb region. Is the other gene (Os11g0609500) expressed in seeds?

Thank you for your comment. In S7 Fig, we illustrated the expression levels of both genes (Os11g0609500 and Os11g0609600) during seed germination. Notably, Os11g0609500 exhibited no expression in seeds.

Line 167: is the other gene sequence identical? The Authors never mention the other gene again.

Thank you for your comment. Only two genes, Os11g0609500 and Os11g0609600 (*GF14h*), were present in the candidate regions. In the case of the other gene (Os11g0609500), a genetic polymorphism also exists between Hitomebore and Arroz da Terra. Nevertheless, as previously mentioned, Os11g0609500 displays no expression in seeds of both Hitomebore and Arroz da Terra. Consequently, the possibility of Os11g0609500 being the candidate gene was ruled out.

Line 193: ‘LTG rates’. It is not a rate.

Thank you for your comment. We have revised the phrasing.

Lines 203-204 in the revised manuscript

…We detected significant drops in the germination percentage in all four knockout lines…

Line 217: The next most common where/among which one? I assume in land races. Please clarify the sentence.

Thank you for your comment. We have revised the phrasing.

Line 230 in the revised manuscript:

The next most common allele combination among the Japanese landraces was…

Line 237: Although I understand the authors, I am not sure that it is correct to write `slightly more susceptible` if the p-value is >0.07. As I found it an important piece of data, I recommend to insert on the main figure.

Thank you for the suggestion. To avoid confusion among readers, we have revised the text as follows (Lines 250-252 in the revised manuscript). And the impact of NIL-*GF14h^Arroz^* on pre-harvest sprouting is not the primary focus of this study; therefore, it will remain as a supplementary figure.

Lines 250-252 in the revised manuscript:

In addition, although no significant difference was observed, it is likely that NIL-*GF14h^Arroz^* tends to be more susceptible to pre-harvest sprouting than Hitomebore (S13 Fig).

Fig 1D: statistical test is needed.

Thank you for your comment. We performed statistical tests.

Fig. 2D: do these data refer to a RT-qPCR? It should be clearly described in the legends.

Thank you for your comment. The expression data presented in Fig 2D was obtained by RT-qPCR. This point is clearly stated in the legends.

Line 815 (Fig 2D legend) in the revised manuscript:

…This expression analysis was conducted by RT-qPCR.

Fig4: it would be interesting to see the germination properties of some of the varieties which have both functional alleles, for example Hokuriku193.

Thank you for your valuable suggestion. We also recognize that the additive or epistatic interaction between *qLTG3-1* and *GF14h* and its effect on LTG are fascinating questions. For future studies, we will explore germination properties across several varieties, including Hokuriku 193.
